# Nursing Challenges in Motivating Nursing Students through Clinical Education: A Grounded Theory Study

**DOI:** 10.1155/2012/161359

**Published:** 2012-07-08

**Authors:** Hanifi Nasrin, Parvizy Soroor, Joolaee Soodabeh

**Affiliations:** ^1^Tehran Nursing and Midwifery school, Tehran University of Medical Sciences (TUMS), Tahran, Iran; ^2^School of Zanjan Nursing and Midwifery, Zanjan University of Medical Sciences (ZUMS), Zanjan, Iran; ^3^Center for Educational Research in Medical Sciences (CERM), Tahran 141973371, Iran

## Abstract

Nurses are the first role models for students in clinical settings. They can have a significant role on students' motivation. The purpose of this study was to explore the understanding of nursing students and instructors concerning the role of nurses in motivating nursing students through clinical education. The sampling was first started purposefully and continued with theoretical sampling. The study collected qualitative data through semistructured and interactive interviews with 16 nursing students and 4 nursing instructors. All interviews were recorded, transcribed, and analyzed using grounded theory approach. One important pattern emerged in this study was the “concerns of becoming a nurse,” which itself consisted of three categories: “nurses clinical competency,” “nurses as full-scale mirror of the future,” and “Monitoring and modeling through clinical education” (as the core variable). The findings showed that the nurses' manners of performance as well as the profession's prospect have a fundamental role in the process of formation of motivation through clinical education. Students find an insight into the nursing profession by substituting themselves in the place of a nurse, and as result, are or are not motivated towards the clinical education.

## 1. Introduction

 One aim of nursing education is to motivate nurses to acquire skills for offering appropriate quality health care services to patients with multiple complex health problems. Achieving this has challenged academic institutions for a long time [1]. 

Studies have shown that, in the clinical setting, the most important barrier to clinical education is students' lack of interest and motivation [2]. Nursing students need long-term motivation to help others in the future [3]. So paying due attention to the concept of motivation is of great importance in clinical education. The environment and human factors are also considered as part of clinical education [4]. Clinical placements form a major part of nursing education and have an important role in students' perceptions of nursing. Nursing students cite unsatisfactory placement experiences as a reason for leaving nursing education [5].

Nurses represent the largest category of health workers and provide 80% of direct patient care [6]. They represent the professional image and the prospect of nursing career and are the main role model for nursing students. Having a clear image of the career helps the students with choosing nursing as a career or retaining it [3, 7, 8].

Some studies suggest that a positive image of nursing may attract applicants [9]. Conversely, a poor public image of nursing contributes to inadequate numbers of students entering the nursing education programmes and, for those that do enter nursing, it can influence the attrition rate [10]. In contrast, Miers et al. found that, although a service orientation remains a key factor in choosing nursing, students also look for a career that matches their interests and attributes, and offers professional values and rewards [3].

Dante et al. explored Italian nursing students' and leavers' perspectives about leaving nursing education. Personal reasons were the most common followed by difficult relationships with teaching staff, learning difficulties, and wrong career choice, as well as difficulties with practical training [11]. Nilsson and Stomberg found that nursing students mainly grade their motivation positively distributed, and different throughout their entire education. The main motivation factor was becoming a nurse. The nursing students mentioned intrinsic motivation factors as explanation for their degree of motivation [12]. Nurses can be regarded as the source of learning at the clinic through social learning pattern [13]. Having substantial teaching background in clinical education, we found a theoretical sensitivity in this area. Hence, considering the gap in the research literature, especially lack of enough studies in this field in Iran, this study was conducted to achieve a deep and comprehensive view and to explore how the nursing students are motivated through clinical education, with an emphasize on the role of nurses in this regard. 

 In Iran, the nursing program offers a four-year Baccalaureate in nursing accredited by the High Council of Medical Education of the Ministry of Health and Medical Education [14]. All schools are obliged to follow a basic curriculum established by the ministry, though some flexibility is allowed within the predetermined curriculum [15]. The program consists of general education courses (20 credits), professional foundation courses (28 credits with a biological content); and nursing courses (53 credits on theory with a biomedical nursing content and 33 credit on clinical preparation). A practicum is an integral part of professional nursing studies and includes a 40-week clinical placement, in which nursing students provide supervised care 7 hours/1day and 6days/week under the supervision of an academic nursing instructor [16].

## 2. Materials and Methods 

### 2.1. Setting and Sample

This paper is part of the first author's Ph.D. dissertation, titled “The process of motivation formation in nursing students during clinical education,” which was conducted using grounded theory method.

Grounded theory focuses on identification, description and explanation of interactional processes among individuals or groups within a given social context [17].

The grounded theory method was selected in this work to explore the participants' opinions on the conditions effective for clinical education. 

In the present research, 16 undergraduate nursing students at the second or higher semesters of study in the Department of Nursing and Midwifery at the Universities of Tehran and Zanjan were selected through purposive sampling. According to their viewpoints and suggestions, it was assumed that nursing instructors serve as suitable key informants regarding clinical education motivation. Considering the wide range of experiences and perceptions among this group of participants, interviews were conducted theoretically according to the codes and categories as they emerged until data saturation. 

Sixteen students and four instructors were invited to participate, and all of them attended an orientation to the study. 

### 2.2. Ethical Considerations

 After approval of the study by the Ethical Committee of the School of Nursing (Tehran University of Medical Sciences) and obtaining permission to proceed from the officials of the Nursing Schools of both Tehran and Zanjan Universities of Medical Sciences, the first author began to collect the data. All participants provided informed consent and all interviews were conducted with prior appointment with the participants. In order to respect the participants' privacy and confidentiality, the interviews were conducted only in the presence of the interviewer and the interviewee. Also, for confidentiality of the information and keeping anonymity, all manuscripts and audio files were coded. All participants were informed that they could withdraw from the study at any time they wished and take their audio files and transcripts; however, this did not happen during the study. 

### 2.3. Data Collection

The method of data collection included semistructured and interactive interviews. The participants chose the location of the interview. The interviews took place at the first author's room at the School of Nursing & Midwifery (Tehran or Zanjan) in a classroom, or in an instructor's room. The length of each interview was between 40 and 120 minutes. The interviews started with broad questions in order to encourage the participants to speak freely and express their personal experiences regarding motivation in clinical education. For example,

*“Can you talk about a typical day at your internship?”, “What conditions influence students' motivation during clinical education?” and “How are nursing students motivated in clinical education?”*

As the interview progressed, the questions became more specific, allowing deeper investigation of the issues raised by the participants in the earlier interviews. The data required were collected and analyzed during a 18-month period from September 2010 until May 2011.

### 2.4. Data Analysis

All interviews were recorded by a digital sound recorder and transcribed verbatim. Then they were managed and sort-coded using MAXQDA 2007 software (VERBI GmbH, Berlin, Germany). The data were analyzed using the constant comparative method. The process of conducting interviews, transcribing the recordings, and analyzing the data occurred simultaneously. In fact, each interview provided direction for the next one. The data collection and analysis continued until data saturation, that is, no additional data were found for development of the category properties. According to the constant comparative analysis, similar data were grouped and conceptually labeled during the so-called “open coding” process. Then the concepts were classified and the categories were linked and organized by relationships in a process, called “axial coding.” Theory integration and refining were conducted by “selective coding” [18]. In this step, core variables emerged as data collection progressed through the constant comparative analysis. The core variable “Monitoring and modeling through clinical education” became a theoretical guide to the further collection and analysis of the data. Therefore, the emerging categories and the core variable led the researcher toward interviewing several key informants who provided rich data about the nurses as role models. This is a repetitious concept and a referral category that can also be generalized to other categories. It further can create linkage among the categories.

### 2.5. Rigor

To maintain trustworthiness, several ways were used. Maximum variant sampling and achievement of saturation were among other items that improved the credibility and the confidentiality of the data, in turn. The process of sampling and analysis of data took 18 months. This prolonged engagement with the participants and their interest helped the investigator to gain the participants' trust and obtain more in-depth data.

For member check, the codes extracted for accuracy of the meanings' interpretation were returned back to all the participants and were approved by them. Credibility and confirmability were established through prolonged engagement, allocation of adequate time, good communication, multiple methods (such as data source triangulation), and maximum variant sampling, which provided plenty of opinions and points of view for the research. The evolving results were discussed continuously among the three researchers as a team. A second review of the transcripts, codes, and grouped codes and concepts by a number of colleagues (as a peer check) and participants (as a member check) was conducted. The majority of colleagues and participants agreed with the initial codes; however, we modified a few codes. In addition, several nursing students who had not participated in the interviews reviewed the results and confirmed their suitability.

### 2.6. Findings

 Ten out of the total of 16 student participants were females and 6 were males, with an average age of 21–25 years. The students were in semesters 4 to 8 of the B.S. course in Nursing. Of the 4 participant instructors, 3 were females and one was male. Their teaching experience ranged from 7 to 30 years. 

In this work, “concerns of becoming a nurse” was emerged as one of the important patterns in the process of motivation formation in the nursing students during clinical education. This pattern consisted of three categories as follows: “nurses clinical competency,” “nurses as full-scale mirror of the future,” and “monitoring and modeling through clinical education” (as the core variable), which will be described in detail in the coming paragraphs. 

#### 2.6.1. Nurses Clinical Competency

In the view of all participants, nurses' clinical competency has been effective on motivation of students in clinical education.

This category comprises three subcategories: (a) caring about patient's rights, (b) nurses clinical knowledge, and (c) submission to routines.

**(a) Caring about Patient's Rights**

In the view of the participants, “due attention of nurses to the spiritual and ideological beliefs of the patient and those accompanying him/her” was among the items of caring about the patient's rights by the nurse. This caring behavior would make the nursing students motivated through their clinical education.

Yet, the participants frequently witnessed cases of not observing the patient's rights by the nurses. The participants listed the aspects of disregard to the patient's rights by the nurses as “misbehavior with the patient,” “not paying attention to physical and psychological needs of the patient,” “not paying attention to the patient's beliefs and/or his/her privacy,” “not preparing the patient,” and “not responding to the patient's questions before conducting any procedure.”

One of the participants spoke about the disregard and inhumane behavior of the nurses toward the patients as follows:

*“For example, even when the patient is screaming of the pain, it happens that the nurse says shut up to the patient. To give a analgesics for the patient is not that difficult but they refrain even from that…They don't consider the patient a human (Participant 7, student).*

As a result, they became pessimistic about nursing, and this in turn decreases their motivation for clinical education.

**(b) Nurses Clinical Knowledge**

Many participants mentioned that nurses' high clinical knowledge leads to their high value, esteem toward them, and acceptance of their suggestions by the physician; all of these improve the students' clinical motivation.

About the influence of the nurses' high levels of clinical knowledge on the motivation of students for clinical education, a participant said:

*“We observe some nurses who are really informed. It is very motivating for the students. For example, I know a nurse who is so educated that can easily comment on the patients' treatment process” (Participant 8, student.) *

Another participant added:

*“A nurse can increase his/her knowledge so that it is possible for him/her to speak with the doctors. It is his/her low level of knowledge that makes him/her stranger to the doctor” (Participant 4, student).*

In contrast to the positive influence of high levels of clinical knowledge, nurses' low clinical knowledge level is among the reasons for physicians, nursing students and patients considering nurses as noneducated. Nurses' low clinical knowledge leads to their “inability to respond to the patient's questions.” As a result, the patient loses confidence on the nurse. 

Another participant stated about the effect of nurses' clinical knowledge on the motivation of student for clinical education:

* “Why do our nurses face such disrespect and distrust? It is because they're not educated…When my student observes an experienced, informed and skilled nurse, he/she would love to the same to receive the same respect. Motivation can be generated in such a way” (Participant 17, instructor).*

A gap between nurses and other professional groups, especially the physicians, would make the participants unmotivated toward the clinical education.

**(c) Submission to Routines**

Submission to routines made the students disinterested in the work in the clinic. Submission to routines was stated as “mechanical care,” “merely giving the drugs prescribed to the patient, taking vital signs, recording and reporting,” “lack of appropriate communication with the patient,” and “lack of teaching the patient.” One of the participants spoke about submission to routines in nurses:

*“You may see in the ward a nurse who has several years of experience but he/she knows nothing and acts just as a robot in giving the drugs, writing the report and handling it to the next shift”( Participant 11, student).*

One of the consequences of submission to routines in nurses is lack of appropriate care for the patients. A participant spoke about this fact:

*“Without seeing to the patient, the nurse sits in the station and writes the nursing report. Student observing such kind of work would definitely wonder why then I should study that much when in the end I would be one like this. Why all this extra burden?” (Participant 9, student).*

These made participants feel that carrying out the nursing tasks needs no knowledge. As a result, they lost their motivation for studying and studentship. 

#### 2.6.2. The Nurse As a Full-Scale Mirror of the Future

The participants believed that students would imagine their future career when observing the work conditions and the nurses' clinical abilities.

This category includes (a) extreme work conditions, and (b) nurses powerlessness.

**(a) Extreme Work Conditions**

In the view of participants, qualified nurses are like a full-scale mirror of the future. Therefore, the work conditions of nurses have a great effect on the students' attitude and motivation. “Extreme work conditions” is an item frequently referred to by the participants. By extreme work conditions, they refer to “disproportionate ratio of nurses to patients”, “job stress and extreme work conditions versus low salary and benefits,” “lack of psychological security,” “carrying out the tasks of other professional groups by nurses,” “having night shifts,” “the hospital's boring environment,” “permanent exposure of the nurses to the disease and death,” and “lack of an appropriate place for the nurses in the clinic and the community.” A participant spoke about the extreme work conditions:

*“When we go for internship, we ask the nurses about their work conditions when we see that they have to work so much for such a low salary. Then we think why we should stand up that much and undergo such a hard work” (Participant 9, student). *

In the view of participants, extreme work conditions made qualified nurses dissatisfied and unmotivated. Consequently, they transferred this dissatisfaction and lack of motivation through their words to the students. Obtaining higher degree, making decision about studying in another field in their M.S. course, or pursuing their study in nursing in order to work in educational environments and thus escaping from the clinical extreme work conditions could be the consequences of the nurses extreme work conditions.

Another student stated that

*“Why do students think of perusing their studies? They are trying to run away from clinical work. Everybody likes convenience. So if possible, they want to engage in education” (Participant 5, student). *

The participants stated that, in the clinical settings, they found no difference between the type of work done by the nurses holding B.S. or M.Sc. degrees. The work of nurses holding M.S. did not differ at all with that of the B.c. nurses or nurse aids. These conditions ultimately result in a decrease of the students' motivation in making efforts to learn and to pursue their studies.

In spite of extreme work conditions in nursing, the participants believed that existing appropriate job market as compared to other majors in Iran will make students more optimistic to the job's future, and to some extent, this is motivating for the students in the clinical education. A participant stated that:

*“The fact that the graduates in other majors are jobless and that it is possible for the nursing students to get in the job market immediately, will be a motivating factor for them” (Participant15, instructor). *

**(b) Nurses Powerlessness**

In the process of physician-nurse communication, the participants observed “a boss-subordinate relation between them” as well as “the nurses' submission to the physicians.” One of the participants believes that

*“Head nurse stands beside the physician. She/he does nothing but to enter into the cardex the physician's orders immediately. What else can we expect? We as students will simply become ones like these after several years…We see the future! So no motivation will be left for the student (Participant 11, student).*

Another student spoke about the fear of nurse in communicating with the physician:

*“The relationship should be so appropriate that the nurse will not be afraid of the physician and ask about every problem he/she encounters. But now it's turned to be so inappropriate that the head nurse even does not dare to ask the physician a single question… She/he does not dare to say that a patient's symptoms have changed” (Participant 14, student).*

Through powerlessness and not being respected, the motivation of nursing students would decrease. In contrast, a good cooperation atmosphere and nurses' self-esteem were encouraging for the students to work in such an environment in the future.

#### 2.6.3. Monitoring and Modeling through Clinical Education

In the present study, “monitoring and modeling through clinical education” emerged to be the core variable. The students monitored and judged on the performance of the nurses and moved on a spectrum of having or not having motivation accordingly. Students frequently substituted nursing roles and imagined themselves in their future jobs. In this respect, a participant stated that:

*“Believe that from now on, we have the concerns of a nurse, and it is as if we are not students at all” (Participant 5, student).*

Through this role replacement, students reach a stage of occupational understanding and cognition, by observing and role replacement of the nurses who have enough clinical knowledge, are not submitted to routines, observe patients' rights, and are innovative in the decrease of clinical nursing problems, the students are motivated for clinical education and use different elements of clinical environment (good communication environment and exploiting learning opportunities) to learn more and better. In contrast, extreme work conditions and nurses' clinical powerlessness can result in decreased motivation of the students in clinical education.

## 3. Discussion

The findings of this study may create a new insight into the relationship of nursing students' challenges in facing the realities of the profession with clinical education motivation. “Concerns of becoming a nurse” was recognized as one of the important patterns of this study. This pattern includes three categories of “nurses clinical competency”, “nurses as full-scale mirror of the future,” and “monitoring and modeling through clinical education” (as the core variable).

Our findings showed that observing moral virtues in nurses as role models motivated the students. Conversely, nonobservation of the patient's rights had a negative effect on the students' motivation. The participants believed that some nurses showed inhuman behavior towards the patients. Witnessing such behaviors, as nursing role models, plus receiving negative feedbacks from the patients about the nurses' behaviors result in a decrease of clinical learning motivation in the students. Recent reports have highlighted negative patient experiences, which reflect a clear lack of compassionate nursing care [19]. In a study conducted by Kyrkjebø and Hage, the participating students experienced the fact that the patients were taken seriously by the staff [20]. In this study, in the participants' view, one of the criteria for clinical competency of nurses was their knowledge. The students, by witnessing the nurses' high clinical knowledge that was materialized in their action, were motivated for continuing their study and clinical work and aspired to act as a knowledgeable nurse in the future. Milisen et al. found that from the participants' perspective, competence seemed to be related to instrumental-technical, intellectual-cognitive, organizational, social and communication skills, and attitudes [21].

Clinical competency promotes skills and authorities of the nurses. Authority itself accompanies with increase of dignity and self-respect in nurses. Such a nurse can be an appropriate model for nursing students and motivate them for clinical learning. In contrast, the nurses with low clinical knowledge made an undesirable impression on the students and, in the opinion of the participants, it had a negative effect on the views of other professional groups in the clinic and, consequently, in the community. In the same context, the study made by Mohammadi et al. showed that more than a half of the nurses in the CCU and ICU units had a weak clinical knowledge [22]. 

The students, when seeing the nurses' submission to routines, refrained from any effort and lost their motivation in learning at the clinic. In submission to routines, a nurse performs his/her predefined duties without any thinking or questioning. In this type of performance, nurses do their duties without paying much attention to their job and, as a results, they have no decision-making power in different situations. Hence, submission to routines takes their authority and power of making decision and adds to the nurses' powerlessness and thus the students' discouragement. 

It is widely accepted that nursing as a career is viewed favorably by the society in that it offers job security, mobility, and career variety [23]. Conversely, in this study, the majority of the students, following the observation of extreme nursing work conditions, sought a way to leave education. Such a student has no motivation for clinical education. In agreement with the findings of this study, in the study of Leong, economic issues were part of the dissatisfaction expressed by the interviewees. Concerns were raised about both salary and employee benefits, and one can conclude that policies in these areas are met with some dissatisfaction [24]. Therefore, imagination of imminent future demotivates nursing students. 

Also, in Jordan, the underpayment of nurses is one of the major reasons for nurses' dissatisfaction and intention to leave hospitals. The lower social status of the nursing profession in terms of low salary drives students away from nursing [25]. 

Elsewhere, a study revealed that wage increases alone did not do much to attract or retain nursing staff: improvement in employment-based benefits was necessary. It was also revealed that the working environment, nurse-patient ratio, and health-care insurance were elements to be considered in the payment and welfare package [26]. 

Powerlessness of nurses in clinic forms a negative view in the mind of students towards the nursing career. Also the study of Brodie et al. showed that high clinical experience of the participant students in this study reinforced both their own and the society's image of an underpaid and overworked profession that lacks respect and has low morale [27]. Nurses' powerlessness has negative impacts on nurses' dignity. Low dignity of the nurses in the clinic decreases clinical learning motivation in the students and represents an unpleasant image from the nursing career to them, because students seek for their future dignity and respect by looking into the present nurses. Health systems should take the promotion of the nurses' dignity into account through providing a dignified work environment [28].

According to the findings of the present research, students who model and substitute nursing roles get insight into their future profession. The findings also showed that nurses as role models had the most influence on the motivation or no motivation of students so that the performance, work conditions and power of nurses each somehow influenced the motivation of students during the clinical education. Many of the strategies used by the students in escaping from clinical environment and future nursing reflect their undesirable evaluation of the nursing profession, which ends with lack of motivation toward clinical education. In return, the participants believed that in Iran proper job market in nursing (compared to the graduated students of other majors who do not find a job easily) can absorb into the health system even those students who were unmotivated during their clinical education. This is in contrast to the research findings in other countries where unmotivated students refrain from entering the job market [25, 27, 29]. The entering of unmotivated students into nursing profession is reflected in the health system of Iran society in facing unmotivated nurses, and each unmotivated student turns into an improper representative of nursing profession. This is how the faulty cycle of creating unmotivated educated nurses continues. Therefore, it is of great importance to pay due attention to strategies for increasing motivation in nursing students. Based on the findings of this study, educational and health care authorities can promote the quality of nurses' work in offering better services to the patients through improving their work conditions. In this way, more appropriate nursing role models would be available for students, and thus their interest in the nursing career would increase. We recommend the conduct of action researches for improvement of the clinical setting in nursing education. 

## 4. Limits of the Present Research 

Although theoretical sampling was used in this work to collect the required data that encompassed multiple aspects of the experience in question, the small sample size were research limitations.
